# SERONEGATIVE NECROLYTIC ACRAL ERYTHEMA: A DISTINCT CLINICAL SUBSET?

**DOI:** 10.4103/0019-5154.70676

**Published:** 2010

**Authors:** S Panda, K Lahiri

**Affiliations:** *From the Department of Dermatology, KPC Medical College, Kolkata, India*; 1 *From the Department of Dermatology, Apollo Gleneagles Hospital, Kolkata, India*

**Keywords:** *Acral erythema*, *hepatitis C*, *necrolytic erythema*, *oral zinc*

## Abstract

A patient was referred to us with asymptomatic, erythematous, nonitchy, scaly lesions present bilaterally on the dorsa of his feet and toes since the last 2 months. Both the legs had pitting edema as well. There were hyperkeratosis, focal parakeratosis, acanthosis and scattered spongiosis in the epidermis, and proliferation of capillaries with perivascular infiltration of lymphomononuclear cells in the dermis. There was no serological evidence of hepatitis C virus. Laboratory investigations revealed hypoalbuminemia and low-normal serum zinc. On clinicopathological correlation, we made a diagnosis of necrolytic acral erythema (NAE). The lesions responded dramatically to oral zinc sulfate and topical clobetasol propionate within 3 weeks with disappearance of edema and scaling and only a minimal residual erythema. This is the first reported case of NAE from Eastern India. NAE with negative serology for hepatitis C may be viewed as a distinct subset of the condition that had been originally described.

## Introduction

Necrolytic acral erythema (NAE) is a newly described entity, first reported by El Darouti and Abu el Ela in 1996 in seven Egyptian patients with hepatitis C virus (HCV).[1] Since then, about 70 cases have been reported from around the world, a great majority of them from Egypt itself, where HCV is hyperendemic.[2] Thus, NAE was recognized as an early cutaneous marker of HCV by virtue of its strong association with this infection.[3] However, a few cases have been reported very recently which were not associated with hepatitis C.[4–6] We report a case of NAE from Eastern India which was seronegative for hepatitis C.

## Case Report

**Figure 1:**
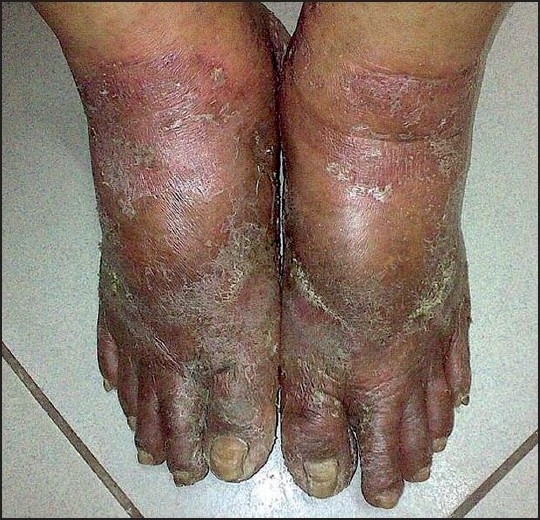
Bilateral, symmetrical, erythematous, scaly lesions involving both feet encroaching the ankles

A 68-year-old man was presented at our clinic with erythematous, nonitchy, scaly lesions manifesting bilaterally and symmetrically on the dorsa of his feet and toes. The lesions extended around the ankles and plantar surfaces, encroaching from the medial aspect [Figure 1].

The rash, which had been present for 2 months before presenting to us, also involved the hands to start with. At the beginning, the areas involved were slightly tender. There was some burning sensation. There was no history of trauma. He was diagnosed initially as having a nonspecific inflammatory dermatosis and was treated with topical and systemic steroids and injection methylcobalamin, before being referred to us. While his hand lesions cleared after initial treatment, those on his lower limbs were unaffected.

The patient had multiple metabolic problems: he was a long-standing diabetic, although under reasonable control (glycosylated hemoglobin-6.1%); he also had hypertriglyceridemia. Interestingly, during last 3 months his serum triglycerides came down from 314.8 mg/dL to 179 mg/dL. The patient was on regular atorvastatin and glipizide for the past few years.

On examination, the patient was found to be a well-built man with skin lesions localized primarily to acral surfaces of his inferior extremities. Well-defined, erythematous, scaly, crusted plaques were seen on both lower legs and feet. The lesions were eroded at places. Both the legs had pitting edema as well. Some linear or elongated ulcerated areas with necrotic debris on the crater were seen with a zone of surrounding erythema around the ankles. The nails were yellowish and brittle with some subungual hyperkeratosis. Interdigital spaces were soggy. Accentuation of the linear contact areas of the footwears (rubber slippers) could be seen on both feet.

The history and clinical examination led us to consider a differential diagnosis of psoriasis (noninvolvement of palms and other extensor surfaces were points going against), eczema (absence of itching at any point of the natural history was the point against), tinea pedis et unguium (bilateral involvement is unusual), NAE (involvement of plantar surface and nails were atypical features) and footwear-contact dermatitis.

On investigation, there was no anemia (hemoglobin-12.2 gm/dL); total leucocyte count and differentials were within normal limits (7600/mm^3^; neutrophils-62%, lymphocytes-29%, monocytes-5%, eosinophils-4%). The erythrocyte sedimentation rate (25 mm/hr) and the C-reactive protein (13 mg/L) were slightly high. Serum albumin was low (3.1 gm/dL). Otherwise, the liver function tests were normal.

Serology for hepatitis B and C were negative. Serum creatinine was normal (0.8 mg/dL). The routine urine examination was noncontributory. The antinuclear antibody was negative. Serum zinc was borderline – 60 mg/dL (normal range: 60-120 mg/dL).

Potassium hydroxide examination of scale scrapings obtained from the affected skin revealed no fungi. Color Doppler study of both lower limb vessels indicated no vascular abnormality. Patch testing with the Indian Standard Battery gave a negative result.

**Figure 2:**
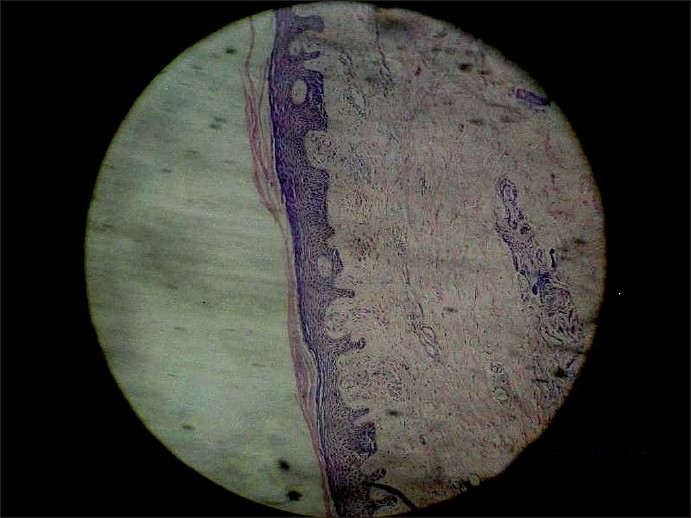
Hyperkeratosis, focal parakeratosis, acanthosis, scattered spongiosis in the epidermis and capillary proliferation with lymphomononuclear infiltration in the dermis

Histopathological examination of a punch biopsy specimen showed hyperkeratosis, focal parakeratosis, acanthosis and scattered spongiosis in the epidermis. Proliferation of capillaries with perivascular infiltration by lymphomononuclear cells was prominent in the dermis [Figure 2]. There was no evidence of vasculitis or hemosiderin-containing macrophages.

**Figure 3:**
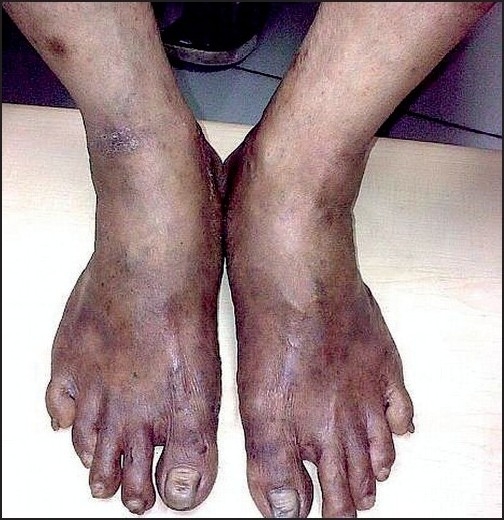
Clinical response after 2 weeks of oral zinc supplementation. There was minimal erythema and practically no scaling and pedal edema

On clinicopathological correlation, a provisional diagnosis of NAE was made and the patient was put on zinc sulfate tablet (200 mg twice daily) and clobetasol propionate cream locally twice daily. After 3 weeks, the response was dramatic. There was a minimal residual erythema. Scaling had virtually vanished. Pedal edema was completely gone. There was only a slight degree of residual hyperkeratosis and some resolving violaceous thinned-out plaques on both lower legs [Figure 3].

Clinicopathological findings as well as a dramatic response to oral zinc supplementation (in spite of a normal zinc level) led us to diagnose the case as NAE. We made this diagnosis in spite of the negative serology for hepatitis C in view of the recent emergence of quite a few seronegative cases of NAE from different parts of the world.[4–6]

## Discussion

Necrolytic acral erythema, after being first described in Egyptian patients with HCV,[1] has been recognized as an early cutaneous marker of that infection. Until now, around 75 cases have been reported in the literature (based on a search of the Pubmed database) from all across the globe, a large majority of these being from Egypt itself (around 65).[3] Although predominantly associated with HCV, in recent times a few cases have been reported (from the United States, Taiwan and India, and interestingly, none from Egypt) without HCV infection. Our case is an addition to the subset of NAE seronegative to HCV.

Other than being HCV-negative, the other atypical features of the case were the involvement of soles and nails and absence of reticular degeneration of superficial keratinocytes with dyskeratosis and vacuolization of basal keratinocytes on microscopy, though there are cases in the literature that have been diagnosed as NAE with these atypical features.[7]

There were several characteristic features including the classic morphology (well-defined, erythematous eruption with marked scaling and a dark red skin), the acral distribution, corroborative laboratory findings (a low serum albumin[8] and low-normal zinc levels[9]) and, last but not least, a dramatic response to oral zinc supplementation in spite of serum zinc levels being within normal limits.[10]

We suggest that in view of the new subset of seronegative NAE being reported from different parts of the world, a therapeutic trial of oral zinc is warranted in patients demonstrating the clinicopathological characteristics of the condition, even in the absence of hypozincemia.
